# Effect of Exposure to Visual Campaigns and Narrative Vignettes on Addiction Stigma Among Health Care Professionals: A Randomized Clinical Trial

**DOI:** 10.1001/jamanetworkopen.2021.46971

**Published:** 2022-02-04

**Authors:** Alene Kennedy-Hendricks, Emma E. McGinty, Amber Summers, Susan Krenn, Michael I. Fingerhood, Colleen L. Barry

**Affiliations:** 1 Department of Health Policy and Management, Johns Hopkins Bloomberg School of Public Health, Baltimore, Maryland; 2 Johns Hopkins Center for Mental Health and Addiction Policy, Baltimore, Maryland; 3 Department of Mental Health, Johns Hopkins Bloomberg School of Public Health, Baltimore, Maryland; 4 Johns Hopkins Center for Communication Programs, Baltimore, Maryland; 5 Division of Addiction Medicine, Johns Hopkins School of Medicine, Baltimore, Maryland

## Abstract

**Importance:**

Stigma toward people with opioid use disorder (OUD) is pervasive in clinical settings, impeding delivery of high-quality care. To date, no study has evaluated the effect of different stigma-reduction messages or messengers among health care professionals.

**Objective:**

To evaluate the effect of OUD-related messages delivered by different messengers on stigma and attitudes toward people with OUD among health care professionals.

**Design, Setting, and Participants:**

This randomized clinical trial examined the effects of OUD-related messages delivered by a visual campaign alone or in combination with a written narrative vignette from the perspective of 1 of 3 messengers. Health care professionals in the US were recruited from 2 national online survey panels (Ipsos KnowledgePanel and SurveyHealthcareGlobus). A total of 1842 participants completed a web-based survey measuring stigma toward people with OUD from November 13 to 30, 2020.

**Interventions:**

Eight groups were exposed to 1 of 2 message frames. One frame (*Words Matter*) emphasized the harm of stigmatizing language, and the other (*Medication Treatment Works*) focused on the effectiveness of medications approved by the US Food and Drug Administration for the treatment of OUD. Message frames were communicated through either a visual campaign alone or a visual campaign in combination with a written narrative vignette from the perspective of a simulated patient with OUD, a clinician, or a health care system administrator.

**Main Outcomes and Measures:**

Dimensions of stigma toward people with OUD were measured on 5-point Likert scales that included items about desire for social distance from people with OUD, perception of individual blame for OUD, perspective of OUD as a medical condition, and support for increased governmental spending on OUD treatment. The level of warmth felt toward people with OUD was measured by a feeling thermometer (range, 0-100 points).

**Results:**

Among 1842 participants, the mean (SD) age was 47 (13) years; 1324 participants (71.9%) were female, 145 (7.9%) were Hispanic, 140 (7.6%) were non-Hispanic Black, 1344 (73.0%) were non-Hispanic White, and 213 (11.6%) were of other non-Hispanic race (ie, individuals who did not self-report race as Black or White and did not self-report ethnicity as Hispanic). Compared with nonexposure, exposure to the combination of visual campaign and narrative vignette communicating the importance of nonstigmatizing language from the perspective of a patient with OUD was associated with a lower probability (difference, −16.8 percentage points, 95% CI, −26.1 to −7.4; *P* < .001) of unwillingness to have a person with OUD marry into the family (a measure of social distance preference) and a 7.2-point (95% CI, 3.2-11.1; *P* < .001) higher warmth rating. Participants exposed to the combined visual campaign and patient vignette about the value of medication treatment for OUD also had significantly lower levels of stigma compared with those in the nonexposed control group (eg, unwillingness to have a person with OUD as a neighbor: difference, −15.3 percentage points; 95% CI, −24.6 to −6.0; *P* = .001).

**Conclusions and Relevance:**

In this study, messages about nonstigmatizing language and effective medication for OUD reduced stigma among health care professionals. Stigma-reduction efforts targeting health care professionals may improve health care system capacity to serve people with OUD.

**Trial Registration:**

ClinicalTrials.gov Identifier: NCT05127707

## Key Points

**Question:**

Are specific communication strategies effective in reducing stigma toward people with opioid use disorder (OUD) among health care professionals?

**Findings:**

In this randomized clinical trial involving a national sample of 1842 health care professionals, exposure to visual campaigns combined with short narrative vignettes told from the perspective of a patient with OUD that emphasized the harm of stigmatizing language or the effectiveness of medications for treating OUD was associated with reduced levels of stigma.

**Meaning:**

The findings of this randomized clinical trial suggest that carefully designed communication campaigns may reduce OUD-related stigma among health care professionals.

## Teaser

This randomized clinical trial assesses the effects of visual campaigns with and without written narrative vignettes on stigma and attitudes toward people with opioid use disorder among a national sample of health care professionals.

## Introduction

Overdose mortality rates have worsened during the SARS-CoV-2 pandemic.^1,2^ Stigma toward people with opioid use disorder (OUD) has been an intractable challenge to ameliorating this problem.^3,4,5^ Defined through a sociological perspective, stigma occurs when the processes of labeling, stereotyping, status loss, and discrimination are enforced through power differentials.^6^ Stigma toward people with OUD and other substance use disorders is rooted in racism, classism, and other systems of oppression.^7,8^ In the health care system, stigma manifests at multiple levels.^9^ Expectation of stigma may reduce treatment seeking and engagement with care.^10,11^ Stigma enacted by clinicians may result in worse quality of care and exacerbate patient mistrust.^4,5,12,13^ On a structural level, stigma contributes to discriminatory policies and reduced investment in systems that support people with OUD.^5,14^ Although interest in stigma-related issues has grown,^3^ the evidence base for stigma-reduction strategies remains limited.^4,15^

Previous studies have reported a connection between language and stigma. Terms such as *addict* and *substance abuser* have been associated with greater stigma relative to person-centered language, such as *person with a substance use disorder*.^16,17,18^ Clinician use of stigmatizing language may translate to worse quality of care.^13^ However, stigmatizing language remains common in clinical and public discourse.^19,20^

In addition to discouraging use of stigmatizing language,^21,22^ communicating about solutions to OUD and overdose, including effective treatment, may mitigate stigma.^4,23,24,25^ Most health care professionals receive minimal training in addiction and its treatment.^26^ Health care professionals may be more aware that they are interacting with patients with OUD when these individuals display OUD symptoms and may be unaware that they are interacting with patients with OUD when these individuals are in recovery,^12^ which could distort the practitioner’s sense of the potential for recovery.

Despite the robust evidence base in favor of medication treatment, stigma has impeded broad availability of highly effective medications approved by the US Food and Drug Administration to treat OUD.^27^ Stigma toward the opioid agonists methadone and buprenorphine is related to the misperception that these medications replace 1 drug for another.^28,29^ Among clinicians, higher levels of stigma have been associated with less interest in prescribing medication for the treatment of OUD.^26,30,31^ Sympathetic narratives that highlight solutions such as effective treatment can help audiences empathize with a person with OUD.^4,15,23,25,32^ Communication strategies focusing on the effectiveness of medication in facilitating recovery may be particularly persuasive to an audience of health care professionals.

Health care professionals are frequently exposed to messages from patients, other clinicians, and health care system administrators. A health care professional may better relate to communication from a fellow health care professional but be more emotionally engaged when messages are delivered by patients. To our knowledge, no study has evaluated the effectiveness of different stigma-reduction messages or messengers among health care professionals. Understanding the ways in which different messages and messengers affect stigma and related attitudes among health care professionals is important to informing the design of effective stigma-reduction communication campaigns in health care settings. In this study, we enrolled a large national sample of health care professionals to assess the impact of 2 message framing strategies communicated through a visual campaign with and without an accompanying written narrative vignette from the perspective of a patient with OUD, a clinician, or a health care system administrator.

## Methods

From November 13 to 30, 2020, we conducted a parallel-group randomized clinical trial involving 1842 health care professionals recruited from a blended sample of 2 online survey panels (Ipsos KnowledgePanel and SurveyHealthcareGlobus). The study was approved by the institutional review board of Johns Hopkins Bloomberg School of Public Health. All participants were members of 1 of the 2 online panels and provided consent to participate in surveys at the time of panel enrollment (with incentives for participation provided by the panel administrators). Therefore, this study was deemed exempt from the need for additional informed consent. The trial protocol was registered retrospectively because the original conception was a survey experiment (Supplement 1). This study followed the Consolidated Standards of Reporting Trials (CONSORT) reporting guideline for randomized clinical trials.

### Data

The study sample included 1242 Ipsos KnowledgePanel members who self-reported their current employment in a health care profession. Ipsos uses probability- and address-based sampling to create an online panel comprising 60 000 members representative of the US population.^33^ Among 2703 KnowledgePanel members invited to participate in the study, 1484 completed the online survey, and 1242 qualified for study inclusion based on current employment in a health care profession (Figure).

**Figure.**
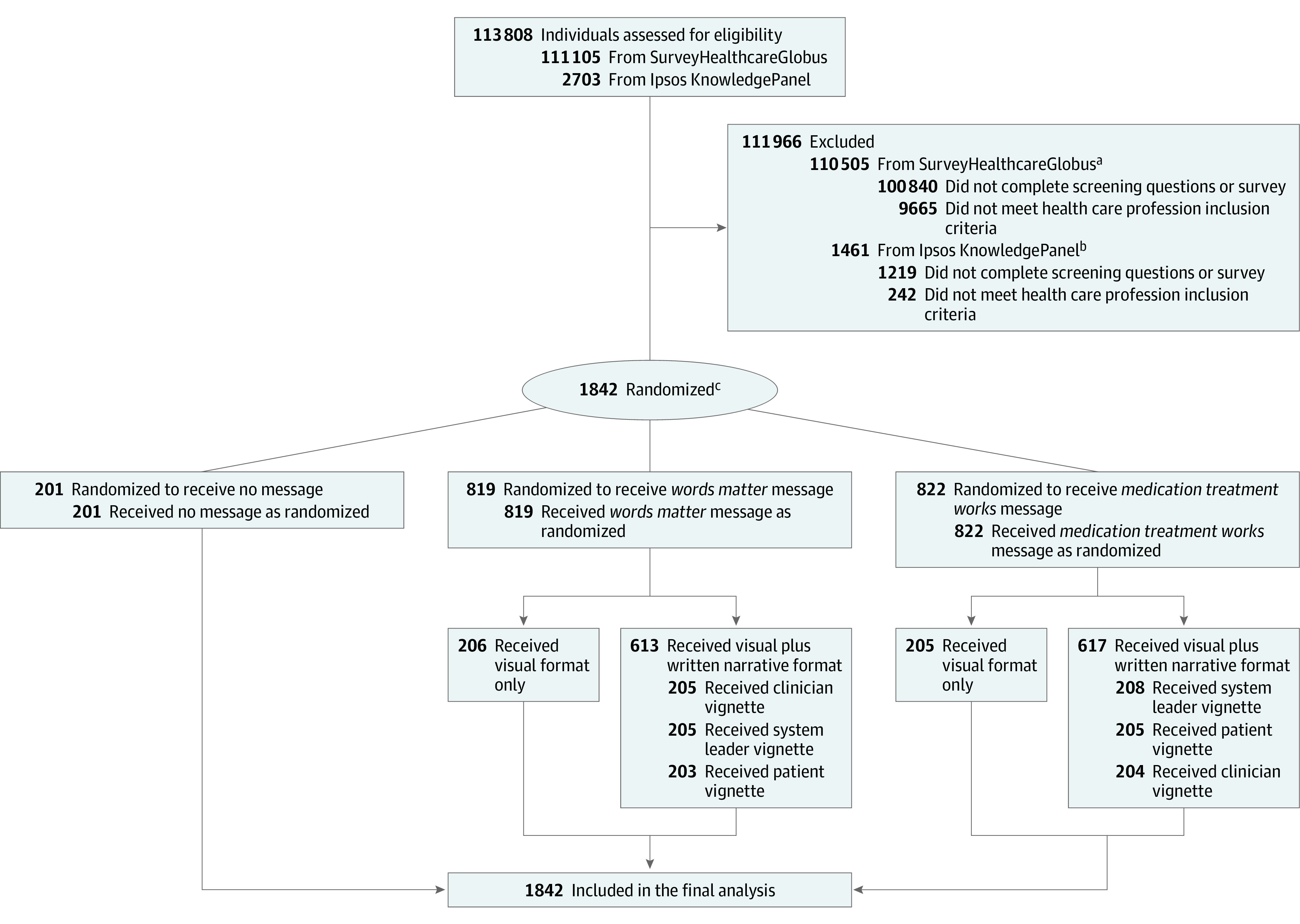
CONSORT Flow Diagram ^a^Excluded if not working in health care profession at time of study. Nurses, physicians categorized as general practitioners or family doctors, and physicians categorized as specialists were included to achieve 600 participants, with approximately one-third represented from each category. ^b^Excluded if not working in health care profession at time of study or if working in a nonqualifying health care profession, such as dentist, dental hygienist, dental assistant, optometrist, veterinarian, veterinary assistant, or massage therapist. ^c^Ipsos KnowledgePanel used a probability proportional to size sampling approach to select 9 random samples.

To ensure greater representation of physicians and nurses, we supplemented the KnowledgePanel sample with 600 members of SurveyHealthcareGlobus, a health care market research firm with an opt-in online panel of physicians and nurses.^34^ Among the 111 105 SurveyHealthcareGlobus panelists invited to participate, 10 265 completed the online survey, and 600 qualified for study inclusion. Our sampling approach was informed by an effort to enroll a large diverse group of health care professionals from across the US. We did not focus on clinicians dedicated to treating OUD because stigma affects quality of care for patients with OUD across all health care settings.

### Study Design

Ipsos used a probability proportional to size sampling approach to select random samples corresponding to 8 exposure groups and 1 nonexposure control group. Each of the 9 groups included approximately 200 participants, providing adequate statistical power to detect medium-sized differences between groups. The 8 groups were exposed to 2 message frames, *Words Matter* and *Medication Treatment Works*, which were communicated through a visual campaign alone or together with a written narrative vignette from the perspective of 1 of 3 messengers: a simulated patient with OUD, a clinician, or a health care system administrator (eTable 1 in Supplement 2). Thus, for each message frame, exposed participants were randomized to 1 of 4 groups: visual campaign only, visual campaign plus patient vignette, visual campaign plus clinician vignette, or visual campaign plus administrator vignette.

The *Words Matter* message frame discouraged use of stigmatizing language related to substance use (including OUD) and encouraged use of nonstigmatizing language. The *Medication Treatment Works* message frame highlighted the value of US Food and Drug Administration–approved medications in facilitating recovery. These message frames were developed during a 2-day communication design workshop held in September 2019 involving 25 invited participants, including individuals receiving treatment for OUD, peer recovery coaches, nurses, physicians, administrators, communication experts, and researchers.

The visual campaigns for the *Words Matter* and *Medication Treatment Works* message frames both displayed headline text stating, “What we say and do matters for patients with substance use disorder,” with images of clinicians interacting with patients (eFigure in Supplement 2). The *Words Matter* visual campaign also displayed stigmatizing terms to avoid along with preferred alternative nonstigmatizing terms and invited participants to take a pledge committing to the use of nonstigmatizing language. The *Medication Treatment Works* visual campaign included text that stated, “Medications help patients recover and live full lives. Don’t let misperceptions get in the way. Learn more about methadone, buprenorphine (also called Suboxone or Subutex), and injectable extended-release naltrexone (also called Vivitrol).”

Participants randomized to receive exposure to the visual campaign in combination with a vignette viewed the visual campaign first, followed by a screen displaying the written narrative vignette. The vignettes were approximately the same length (165 words) and communicated the message frame from the perspectives of the 3 different messengers (patient with OUD, clinician, or health care system administrator) (eTable 2 in Supplement 2). For instance, in the patient vignette for the *Words Matter* message frame, the patient described interacting with clinicians using language such as *addict* and feeling as though the clinicians “didn’t see me as a person” and “all they could see was my addiction.” In the clinician vignette, the clinician described using terms such as *addict* and learning that this language made patients feel as though “I didn’t see them as people” and “all I could see was their addiction.” In each narrative vignette, the patient, clinician, and health care system administrator evolved in their understanding of the harm of stigmatizing language, and the vignettes ended by noting that “health care professionals can be role models.” The *Medication Treatment Works* vignettes included similar content and language across the 3 different messenger narratives and shared themes with the *Words Matter* message frames, including an emphasis on health care professionals as role models. The *Medication Treatment Works* patient vignette featured a person recovering from OUD with the help of medication; the clinician and administrator vignettes described positive experiences with providing medication for the treatment of patients with OUD.

### Measures

After exposure to the visual campaign with or without an accompanying narrative vignette, all participants answered questions designed to measure stigma toward people with OUD (domain A), the primary set of outcomes. Participants in the control group answered the questions with no preceding exposure to either message frame. After completing questions for domain A, participants randomized to 1 of the 4 *Words Matter* groups answered questions about the appropriateness of various terms (domain B). Participants randomized to 1 of the 4 *Medication Treatment Works* groups answered questions about OUD medication treatment (domain C). Participants randomized to the control group answered questions in all 3 domains. For all participants, domain A questions appeared first; for the control group, the order of domains B and C was randomized. The order of questions within each domain was randomized for all groups to reduce risk of bias due to priming.

Domain A included items adapted from the General Social Survey stigma module^35,36^ and from published scales measuring stigma.^14,30^ These items assessed preferences for social distance (such as unwillingness to have a person with OUD marry into their family or be a neighbor),^14,30,36,37^ perspectives on the causes of OUD,^14,30,36^ support for increased governmental spending on OUD programs, and level of warmth felt toward people with OUD.^38^ Dimensions of stigma were measured using 5-point Likert scales, and levels of warmth were measured by a feeling thermometer (range, 0-100 points).

Domain B questions asked participants to rate on a 5-point Likert scale the extent to which they agreed or disagreed that language was appropriate to use in clinical settings. The language included 5 stigmatizing terms (*addict*, *substance abuse*, *dirty* and *clean* [in reference to drug test results], and *addicted baby*) and 5 alternative nonstigmatizing terms (*person with substance use disorder*, *substance use*, *negative* and *positive* [in reference to drug test results], and *baby born with neonatal opioid withdrawal syndrome*). We selected terms based on previous work suggesting that these terms elicited different levels of stigma.^16,17,18^ We created a stigmatizing terms scale (Cronbach α = .74) by summing the Likert scale responses to the stigmatizing terms and dividing by the total number of items. We used the same procedure for the alternative terms to create a nonstigmatizing terms scale (Cronbach α = .72). Participants in the *Words Matter* and control groups reported whether they would sign a *Words Matter* pledge committing to the use of nonstigmatizing language.

Domain C began by asking participants whether a treatment for OUD existed that was effective over a long period.^39^ This question was followed by a short explanation of the Food and Drug Administration–approved medications for the treatment of OUD. Participants then responded to adapted versions of several questions from domain A (substituting “a person taking medication to treat OUD” for “a person with OUD”) and questions about perceived effectiveness of OUD medication treatment^26,30,31^ (all survey questions are available in eTable 3 in Supplement 2).

### Statistical Analysis

We used unpaired 2-sided *t* tests and χ^2^ tests to assess differences in observed characteristics across study groups. We dichotomized Likert scale responses (eg, *agree* and *strongly agree* were coded as 1, and other responses were coded as 0) and estimated logistic and linear regression models (for the feeling thermometer item and scales) to assess differences in responses between the exposure groups and the control group. In the models, we controlled for participant age, sex, prescriber status (physician, nurse practitioner, or physician assistant), and race and ethnicity (non-Hispanic White or non-Hispanic other race). We calculated predicted margins and marginal effects to display results for binary outcomes as predicted probabilities and changes in predicted probabilities. To correct for multiple hypothesis testing, we generated sharpened false discovery rate q values and identified estimates with q values less than .05 as statistically significant (with 2-sided *P* values reported to aid interpretation of significance).^40^ To estimate the marginal effect of the narrative vignettes on outcomes, we also assessed differences between groups exposed to both the visual campaign and a narrative vignette vs those exposed to the visual campaign only. In a sensitivity analysis, we estimated ordered logistic regression models using the nondichotomized Likert scale responses. Data were analyzed using Stata software, version 14 (StataCorp LLC).

## Results

### Participants

Among 1842 participants, 1324 individuals (71.9%) were female and 518 (28.1%) were male. With regard to race and ethnicity, 145 participants (7.9%) were Hispanic, 140 (7.6%) were non-Hispanic Black, 1344 (73.0%) were non-Hispanic White, and 213 (11.6%) did not self-report race as Black or White and did not self-report ethnicity as Hispanic. Most participants were nurses (505 individuals [27.4%]) or physicians (467 individuals [25.4%]) (Table 1).

**Table 1.** Participant Characteristics

Characteristic	Participants, No. (%)
Total participants, No.	1842
Age, mean (SD), y	47 (13)
Educational level	
High school or less	87 (4.7)
Some college	431 (23.4)
Bachelor's degree or higher	1324 (71.9)
Sex	
Female	1324 (71.9)
Male	518 (28.1)
Race and ethnicity	
Hispanic	145 (7.9)
Non-Hispanic Black	140 (7.6)
Non-Hispanic White	1344 (73.0)
Non-Hispanic other race^a^	213 (11.6)
Annual household income, $	
<50 000	261 (14.2)
50 000-99 999	560 (30.4)
100 000-149 999	405 (22.0)
150 000-199 999	215 (11.7)
>200 000	401 (21.8)
Married or living with partner	1285 (69.8)
Household size	
1	349 (18.9)
2	621 (33.7)
3	323 (17.5)
≥4	549 (29.8)
Owns home	1505 (81.7)
Lives in metropolitan area	1622 (88.1)
Region of US	
South	639 (34.7)
Midwest	512 (27.8)
West	346 (18.8)
Northeast	345 (18.7)
Health care profession	
Registered nurse or licensed practical nurse	505 (27.4)
Physician	467 (25.4)
Health aide or assistant	238 (12.9)
Health technician or technologist	219 (11.9)
Nurse practitioner or physician assistant	116 (6.3)
Therapist	106 (5.8)
Other practitioner	191 (10.4)

^a^ Includes individuals who did not self-report race as Black or White and did not self-report ethnicity as Hispanic.

A total of 201 participants were randomized to the nonexposed control group, 206 to the *Words Matter* visual campaign–only group, 203 to the *Words Matter* visual campaign plus patient vignette group, 205 to the *Words Matter* visual campaign plus clinician vignette group, and 205 to the *Words Matter* visual campaign plus administrator vignette group. A total of 205 participants were randomized to the *Medication Treatment Works* visual campaign–only group, 205 to the *Medication Treatment Works* visual campaign plus patient vignette group, 204 to the *Medication Treatment Works* visual campaign plus clinician vignette group, and 208 to the *Medication Treatment Works* visual campaign plus administrator vignette group (Figure). We observed no significant differences in participant characteristics across study groups (eg, 133 participants [66.2%] in the control group, 585 participants [71.4%] in the *Words Matter* group, and 604 participants [73.5%] in the *Medication Treatment Works* group were female; 64 participants [31.8%] in the control group, 270 participants [33.0%] in the *Words Matter* group, and 249 participants [30.3%] in the *Medication Treatment Works* group were prescribing clinicians) (eTable 4 in Supplement 2).

### Attitudes Toward People With Opioid Use Disorder

In the control group, 142 participants (70.6%) reported unwillingness to have a person with OUD marry into their family, and 90 participants (44.8%) reported unwillingness to have a person with OUD as a neighbor (Table 2). Most participants in the control group disagreed that people with OUD had only themselves to blame for the problem (118 individuals [58.7%]) and favored increased spending on OUD treatment (131 individuals [65.2%]). The mean (SD) level of warmth toward people with OUD was 50.7 (19.1) points on the feeling thermometer scale.

**Table 2.** Effect of Exposure vs Nonexposure to *Words Matter* and *Medication Treatment Works* Message Frames on Stigma Toward People With Opioid Use Disorder^a^

Survey item	Control group	Percentage point differences in attitudes or differences in warmth between exposure group and control group (95% CI)															
		Words Matter message frame								Medication Treatment Works message frame							
		Visual campaign only	*P* value	Visual campaign + patient vignette	*P* value	Visual campaign + clinician vignette	*P* value	Visual campaign + administrator vignette	*P* value	Visual campaign only	*P* value	Visual campaign + patient vignette	*P* value	Visual campaign + clinician vignette	*P* value	Visual campaign + administrator vignette	*P* value
Attitudes toward people with OUD (percentage point difference)																	
Unwilling to have person with OUD marry into family	142 (70.6)	0.3 (–8.5 to 9.1)	.94	–16.8 (–26.1 to –7.4)^b^	<.001	–10.2 (–19.4 to –1.0)	.03	–6.6 (–15.7 to 2.5)	.15	–5.3 (–14.3 to 3.7)	.25	–14.5 (–23.8 to –5.3)^b^	.002	–7.0 (–16.1 to 2.1)	.13	–8.8 (–17.9 to 0.30)	.06
Unwilling to have person with OUD as neighbor	90 (44.8)	–0.6 (–10.2 to 9.1)	.91	–12.2 (–21.7 to –2.8)	.01	–6.2 (–15.8 to 3.4)	.20	–8.0 (–17.6 to 1.5)	.10	–5.3 (–14.9 to 4.3)	.28	–15.3 (–24.6 to –6.0)^b^	.001	–8.2 (–17.7 to 1.3)	.09	–9.5 (–18.9 to –0.1)	.047
Agree OUD is a medical condition	108 (53.7)	6.2 (–3.2 to 15.6)	.20	6.2 (–3.1 to 15.6)	.19	5.6 (–3.9 to 15.2)	.25	9.3 (0 to 18.7)	.049	3.1 (–6.3 to 12.4)	.52	5.9 (–3.5 to 15.3)	.22	4.3 (–5.1 to 13.7)	.37	7.0 (–2.4 to 16.3)	.15
Disagree people with OUD are to blame	118 (58.7)	–7.7 (17.3 to 1.8)	.11	–1.7 (–11.2 to 7.9)	.74	–5.7 (–15.4 to 3.9)	.24	1.9 (–7.6 to 11.1)	.70	–6.5 (–15.9 to 3.0)	.18	–1.5 (–11.0 to 8.1)	.76	–1.0 (–10.4 to 8.6)	.85	–4.8 (–14.2 to 4.7)	.33
Favor increased spending on OUD treatment	131 (65.2)	–2.9 (–12.2 to 6.4)	.54	6.1 (–2.9 to 15.1)	.18	–1.5 (–10.8 to 7.8)	.75	2.0 (–7.1 to 11.1)	.67	–2.7 (–11.9 to 6.6)	.57	1.3 (–7.9 to 10.4)	.79	3.7 (–5.3 to 12.8)	.42	1.9 (–7.3 to 11.1)	.68
Warmth toward people with OUD, mean (SD) (level of difference)^c^	50.7 (19.1)	–0.3 (–4.4 to 3.8)	.89	7.2 (3.2 to 11.1)^b^	<.001	1.2 (–3.2 to 4.7)	.72	5.6 (1.6 to 9.6)^b^	.006	3.0 (–1.1 to 7.1)	.16	6.4 (2.3 to 10.5)^b^	.002	1.8 (–2.1 to 5.7)	.37	5.9 (2.0 to 9.7)^b^	.003

Abbreviation: OUD, opioid use disorder.

^a^ Analysis included all 1842 participants. Logit regression models were used to estimate differences between exposure groups and nonexposure control group. Percentage point differences and mean differences in warmth level were calculated using postestimation marginal effects. Model estimates were adjusted for age (continuous), female sex, prescriber status (physician, nurse practitioner, or physician assistant), and race and ethnicity (non-Hispanic White or non-Hispanic other race).

^b^ Estimate reached threshold of statistical significance as defined by a sharpened false discovery rate q value of <.05. Sharpened q values rather than *P* values were used to correct for multiple hypothesis testing.

^c^ Score range, 0-100 points.

Exposure to the *Words Matter* and *Medication Treatment Works* visual campaigns alone was not associated with statistically significant differences from the control group (eg, unwilling to have a person with OUD marry into the family: difference, 0.3 percentage points [95% CI, −8.5 to 9.1; *P* = .94] in the *Words Matter* group and −5.3 percentage points [95% CI, −14.3 to 3.7; *P* = .25] in the *Medication Treatment Works* group). However, exposure to the combined *Words Matter* visual campaign and patient vignette was associated with a lower probability of unwillingness to have a person with OUD marry into the family (difference, −16.8 percentage points; 95% CI, −26.1 to −7.4; *P* < .001) and a 7.2-point (95% CI, 3.2-11.1; *P* < .001) higher warmth rating than nonexposure. Exposure to the combined *Medication Treatment Works* visual campaign and patient vignette was associated with lower probabilities of unwillingness to have a person with OUD marry into the family (difference, −14.5 percentage points; 95% CI, −23.8 to −5.3; *P* = .002) and to have a person with OUD as a neighbor (difference, −15.3 percentage points; 95% CI, −24.6 to −6.0; *P* = .001) as well as a 6.4-point (95% CI, 2.3-10.5; *P* = .002) higher warmth rating than nonexposure.

Exposure to the combined *Words Matter* visual campaign and administrator vignette was associated with a 5.6-point (95% CI, 1.6-9.6; *P* = .006) higher warmth rating than nonexposure. Exposure to the combined *Medication Treatment Works* visual campaign and administrator vignette was associated with a 5.9-point (95% CI, 2.0-9.7; *P* = .003) higher warmth rating than nonexposure. For both message frames, exposure to the combined visual campaign and clinician vignette was not associated with significantly different levels of stigma compared with nonexposure.

Relative to exposure to the visual campaign alone, exposure to the combined visual campaign and patient vignette was associated with lower levels of stigma in both the *Words Matter* and *Medication Treatment Works* groups (eg, unwilling to have a person with OUD as a neighbor: difference, –12.1 percentage points [95% CI, –21.5 to –2.8; *P* = .01] in the *Words Matter* group and –9.9 percentage points [95% CI, –19.0 to –0.7; *P* = .04] in the *Medication Treatment Works* group) (eTable 5 in Supplement 2).

### Stigmatizing Language and Alternative Terms

In the control group, 93 participants (46.3%) agreed that *addict* was an appropriate term to use in a clinical setting, and 163 participants (81.1%) agreed that *substance abuse* was an appropriate term (eTable 6 in Supplement 2). A total of 148 participants in the control group (73.6%) were willing to sign a pledge to use nonstigmatizing language.

Compared with nonexposure, exposure to the *Words Matter* visual campaign combined with the patient vignette was associated with lower probabilities of endorsing *addict* (difference, −23.1 percentage points; 95% CI, −32.1 to −14.2; *P* < .001), *substance abuse* (difference, −23.3 percentage points; 95% CI, −31.9 to −15.0; *P* < .001), *dirty* (difference, −9.0 percentage points; 95% CI, −15.7 to −2.3; *P* = .009), and *clean* (difference, −22.9 percentage points; 95% CI, −33.2 to −13.5; *P* < .001) as acceptable terms as well as a 0.5-point lower rating (95% CI, −0.7 to −0.4; *P* < .001) on the 5-point scale measuring perceived acceptability of stigmatizing terms. We observed similar reductions among those exposed to the visual campaign combined with the clinician vignette (eg, endorsement of *addict* as an appropriate term: difference, −16.4 percentage points; 95% CI, −25.6 to −7.1; *P* = .001) or the administrator vignette (eg, endorsement of *addict* as an appropriate term: difference, −20.4 percentage points; 95% CI, −29.5 to −11.3; *P* < .001) compared with the control group. None of the exposures was associated with a greater willingness to sign a pledge to use nonstigmatizing language (visual campaign only: difference, −3.0 percentage points [95% CI, −15.4 to 9.4; *P* = .63]; visual campaign plus patient vignette: difference, 0.4 percentage points [95% CI, −7.9 to 8.8; *P* = .92]; visual campaign plus clinician vignette: difference, −1.6 percentage points [95% CI, −23.0 to 2.0; *P* = .10]; visual campaign plus administrator vignette: difference, 3.4 percentage points [95% CI, −4.7 to 11.6; *P* = .41]) compared with the control group. The *Words Matter* visual campaign and vignette combinations were associated with greater recognition of stigmatizing language than the visual campaign alone (eg, endorsement of *addict* as an appropriate term: difference, −18.5 percentage points [95% CI, −27.3 to −9.7; *P* < .001) for visual campaign plus patient vignette, −11.8 percentage points [95% CI, −20.9 to −2.6; *P* = .01] for visual campaign plus clinician vignette, and −15.7 percentage points [95% CI, −24.7 to – 6.8; *P* < .001] for visual campaign plus administrator vignette) (eTable 7 in Supplement 2).

### Attitudes About Medications for Opioid Use Disorder

The *Medication Treatment Works* visual campaign combined with the patient vignette was associated with a 4.6-point (95% CI, 0.3-9.0; *P* = .04) higher warmth rating toward people receiving medication to treat OUD, with a similarly higher warmth rating (difference, 4.4 points; 95% CI, 0.2-8.6; *P* = .04) among those exposed to the combined visual campaign and administrator vignette (eTable 8 in Supplement 2). However, exposure to the *Medication Treatment Works* message frames was not associated with any other significant differences in attitudes related to medication treatment compared with nonexposure (eg, unwillingness to have a person receiving OUD medication treatment as a neighbor: difference, −5.3 percentage points; 95% CI, −12.8 to 2.3; *P* = .17). Sensitivity analyses estimating ordered logistic regression models with nondichotomized Likert scale response options produced qualitatively similar results (eTable 9 to eTable 11 in Supplement 2).

## Discussion

This randomized clinical trial assessed the effects of 2 message frames delivered through visual campaigns and written narrative vignettes from the perspectives of a patient with OUD, a clinician, or a health care system administrator among a large national sample of health care professionals. Our findings suggested that visual campaigns alone may have limited impact on stigma toward people with OUD. A potentially more effective communication strategy may combine these visual campaigns with narrative vignettes featuring the voices of people with OUD.

Findings were consistent with other research suggesting that messages emphasizing nonstigmatizing language^16,17,18^ and highlighting effective treatment^23,25^ through sympathetic stories^23,25,32^ can reduce stigma among the public and that narrative-based messaging can be an effective means of communicating important information to clinicians.^41^ Both the *Words Matter* and *Medication Treatment Works* message frames were associated with lower levels of stigma when they incorporated vignettes, particularly vignettes featuring narratives from the perspective of a patient with OUD. Exposure to narrative vignettes from all messengers (patients, clinicians, and administrators) was associated with greater recognition of stigmatizing language. Health care systems seeking to reduce stigma may consider highlighting these voices in their communication efforts. Hiring and integrating people with lived experience into the health care professional workforce is a contact-based approach that may also be an effective stigma-reduction strategy.

Several high-profile communication campaigns have used images and stories of real individuals with OUD who received treatment with medication.^42,43^ Notably, our *Medication Treatment Works* vignettes were not associated with lower levels of medication-related stigma. Other strategies are likely needed to reduce stigma toward these highly effective medications, including structural and policy reforms to improve access.^44^ Several health care systems have implemented campaigns to discourage use of stigmatizing language and encouraged staff to sign language pledges.^21,22^ Although the results of the present study suggest that these pledges have the potential to improve recognition of stigmatizing terms that are inappropriate for use in clinical settings, we did not find that the *Words Matter* message frame was associated with greater willingness to sign a language pledge. Communication campaigns likely need to be implemented together with other interventions to change behavior rather than knowledge and attitudes only.

Stigma toward people with OUD and other substance use disorders has been constraining our ability to resolve the persistent drug overdose problem.^5^ Clinical settings are meant to be welcoming and safe environments in which people with substance use disorders can seek care and connect to services. Given that stigma among health care professionals can have serious consequences for patients with OUD, stigma-reduction communication campaigns are needed to target this population. Encouraging changes in language and improving understanding of the effectiveness of medication treatment may reduce stigma toward people with OUD.

### Limitations

This study has several limitations. First, although samples were recruited from 2 national online survey panels, those panels are not probability-based samples of health care professionals, which may affect generalizability. Sample characteristics indicate geographically diverse representation from a variety of health care professions. Although 71.9% of participants were female, this percentage is consistent with the composition of the broader health care professional workforce.^45^ Second, we did not use vignettes from real individuals, although message design was informed by input from health care professionals and people with lived experience. We constructed the exposures to minimize confounding and enhance our ability to isolate the impact of the most important elements of the messages and messengers. When designing campaigns for health care systems, the inclusion of real people in communication materials could increase the authenticity of the campaign; whether this inclusion changes the effects of these messages is unknown. Third, participants answered questions directly after viewing the visual campaign and/or vignettes a single time. The durability of effects and the impact of repeat exposure (during an extended campaign) are uncertain. Fourth, we conducted the study during the SARS-CoV-2 pandemic, which strained the health care workforce, potentially affecting responses. Fifth, we could not translate estimated changes in attitudes into measures of clinical impact; future work is warranted to explore these connections.

## Conclusions

In this randomized clinical trial, messages about nonstigmatizing language and effective medication for the treatment of OUD were associated with reductions in stigma toward people with OUD among health care professionals. Stigma-reduction efforts targeting health care professionals may improve health care system capacity to serve people with OUD.

## Notes

Supplement 1.
Trial Protocol

Supplement 2. **eTable 1.** Structure of Randomized Clinical Trial **eTable 2.** Narrative Vignettes Communicating *Words Matter* and *Medication Treatment Works* Message Frames **eTable 3.** Questions Included in Each Domain **eTable 4.** Comparison of Characteristics of Survey Participants Across Study Arms **eTable 5.** Effects of Exposure to the *Words Matter* and *Medication Treatment Works* Narrative Vignette and Visual Campaign Combination on Stigma Toward People With Opioid Use Disorder Relative to the Visual Campaign–Only Groups **eTable 6.** Effects of Exposure to the “Words Matter” Visual Campaign and Vignette Frames on Perceptions of Clinically Appropriate Language Related to Substance Use Relative to the No-Exposure Control Group **eTable 7.** Effects of Exposure to the *Words Matter* Narrative Vignette and Visual Campaign Combination on Perceptions of Clinically Appropriate Language Related to Substance Use Relative to the Visual Campaign–Only Group **eTable 8.** Effects of Exposure to the “Medication Treatment Works” Visual Campaign and Vignettes on Stigma toward People with Opioid Use Disorder Receiving Medication Treatment Relative to the No-Exposure Control Group **eTable 9.** Effects of Exposure to the *Words Matter* and *Medication Treatment Works* Message Frames on Stigma Toward People With Opioid Use Disorder Relative to the Nonexposed Control Group Estimated With Ordinal Logistic Regression Models **eTable 10.** Effects of Exposure to the *Words Matter* Narrative Vignette and Visual Campaign Combination on Perceptions of Clinically Appropriate Language Related to Substance Use Relative to the Visual Campaign–Only Group Estimated With Ordinal Logistic Regression Models **eTable 11.** Effects of Exposure to the *Medication Treatment Works* Visual Campaign and Narrative Vignettes on Stigma Toward People With Opioid Use Disorder Receiving Medication Treatment Relative to the Nonexposed Control Group Estimated With Ordinal Logistic Regression Models **eFigure.** *Words Matter* and *Medication Treatment Works* Visual Campaigns

Supplement 3.
Data Sharing Statement
