# Supplementary material for: Effect of Exposure to Visual Campaigns and Narrative Vignettes on Addiction Stigma Among Health Care Professionals: A Randomized Clinical Trial
Source: JAMA Netw Open. 2022 Feb 4;5(2):e2146971. doi: 10.1001/jamanetworkopen.2021.46971 (PMC8817201; doi:10.1001/jamanetworkopen.2021.46971)
Supplement: Supplement 1. — Trial Protocol [file jamanetwopen-e2146971-s001.pdf]

Strategies to Reduce Addiction Stigma Among Health Professionals Randomized Experiment Protocol

Design
This study was a parallel group randomized survey experiment. Participants answered questions in a survey following either no exposure (control group) or exposure to one of eight messages communicated through visual or visual combined with narrative vignettes communicating messages intended to reduce stigma toward people with opioid use disorder.

Study Population
Study participants included 1,842 U.S. adults currently working in a health profession and serving as panelists on one of two online survey panels, which composed the sampling frame.

Inclusion Criteria
Adult with current employment in a health profession in the U.S. who were panelists in Ipsos probability-based KnowledgePanel or SurveyHealthcareGlobus's opt-in panel of physicians and nurses during November 2020 when this survey experiment was fielded.

Exclusion Criteria
Not currently working in a health profession at the time of the study or employed in a non-qualifying health profession, including dentist, dental hygienist, dental assistant, optometrist, veterinarian, veterinary assistant, or massage therapist.

Randomization
Ipsos used a probability-proportional-to-size sampling approach to select nine random samples corresponding to the nine study arms.

Intervention/Exposure
Participants in the experimental groups were exposed to one of eight stigma reduction message frames (the remaining arm was a no-exposure control group). The eight exposures included:
(1) a visual displaying information on stigmatizing language to avoid in clinical settings related to substance use disorder and non-stigmatizing alternative language;
(2) a visual displaying information on stigmatizing language to avoid in clinical settings related to substance use disorder and non-stigmatizing alternatives language and a narrative vignette told from the perspective of a patient with opioid use disorder about the importance of non-stigmatizing language in clinical settings;
(3) a visual displaying information on stigmatizing language to avoid in clinical settings related to substance use disorder and non-stigmatizing alternatives language and a narrative vignette told from the perspective of a clinician about the importance of non-stigmatizing language in clinical settings;
(4) a visual displaying information on stigmatizing language to avoid in clinical settings related to substance use disorder and non-stigmatizing alternatives language and a narrative vignette told from the perspective of a health system administrator about the importance of non-stigmatizing language in clinical settings;
(5) a visual displaying information on the effectiveness of the FDA-approved medications in treating opioid use disorder;

(6) a visual displaying information on the effectiveness of the FDA-approved medications in treating opioid use disorder and a narrative vignette told from the perspective of a patient with opioid use disorder about their successful treatment involving medication;
(7) a visual displaying information on the effectiveness of the FDA-approved medications in treating opioid use disorder and a narrative vignette told from the perspective of a clinician about the effectiveness of medication treatment of opioid use disorder;
(8) a visual displaying information on the effectiveness of the FDA-approved medications in treating opioid use disorder and a narrative vignette told from the perspective of a health system administrator about the effectiveness of medication treatment of opioid use disorder.

Primary Outcomes
The primary set of outcomes included participants' responses to survey questions intended to measure of several dimensions of stigma, including: preferences for social distance from people with opioid use disorder; perceptions of the cause and blame for opioid use disorder; support for government spending on opioid use disorder; and warmth felt for people with opioid use disorder. The specific survey items included the following:

(1) Willingness to have a person with opioid use disorder marry into your family, assessed on a 5-point Likert scale from strongly willing to strongly unwilling
(2) Willingness to have a person with opioid use disorder as a neighbor, assessed on a 5-point Likert scale from strongly willing to strongly unwilling
(3) Agreement with the statement "Opioid use disorder is a chronic medical condition like diabetes mellitus," measured on a 5-point Likert scale ranging from strongly agree to strongly disagree
(4) Agreement with the statement "Individuals with opioid use disorder have only themselves to blame for their problem," measured on a 5-point Likert scale ranging from strongly agree to strongly disagree
(5) Favor for increasing government spending on treatment for opioid use disorder, measured on a 5-point Likert scale ranging from strongly oppose to strongly favor
(6) Warmth felt toward people with opioid use disorder, measured using a 0-100 scale feeling thermometer.

Secondary Outcomes
Two secondary sets of outcomes included ratings of appropriateness of various terms in clinical settings and perceptions of medication treatment for opioid use disorder:

<u>Ratings of appropriateness of language in clinical settings</u> - Ratings on 5-point Likert scales ranging from strongly agree to strongly disagree of agreement that each of the below terms is appropriate in a clinical care setting:
(1) addict
(2) substance abuse
(3) dirty in reference to a drug test result
(4) clean in reference to a drug test result
(5) addicted baby
(6) person with substance use disorder
(7) substance use

(8) negative in reference to a drug test result
(9) positive in reference to a drug test result
(10) baby born with neonatal opioid withdrawal syndrome

Yes/no willingness to sign a "Words Matter" pledge committing to using non-stigmatizing language about substance use disorder when working in clinical settings.

<u>Perceptions of medication treatment for opioid use disorder</u>
(1) Belief that there is a treatment for opioid use disorder that is effective for a long period of time (response options: yes, no, don't know)
(2) Willingness to have a person taking medication to treat opioid use disorder marry into your family, assessed on a 5-point Likert scale from strongly willing to strongly unwilling
(3) Willingness to have a person taking medication to treat opioid use disorder as a neighbor, assessed on a 5-point Likert scale from strongly willing to strongly unwilling
(4) Rating of people taking medication to treat opioid use disorder as weak or strong on a 5-point Likert scale
(5) Agreement with statement "Medication treatment for opioid use disorder is more effective than treatment without medication," measured on a 5-point Likert scale ranging from strongly agree to strongly disagree
(6) Agreement with statement "Most people with opioid use disorder will, with medication treatment, get well and return to productive lives," measured on a 5-point Likert scale ranging from strongly agree to strongly disagree
(6) Warmth felt toward people taking medication to treat opioid use disorder, measured using a 0-100 scale feeling thermometer.

Data Collection and Follow-Up
Data was collected immediately after exposure to the visual and/or visual combined with narrative vignettes via survey questionnaire. There was no follow-up.

Statistical Approach
Descriptive statistics were used to characterize the study population and each experiment arm. Logistic regression models were used to assess differences between each experimental group and the no-exposure group using dichotomized Likert scale items. Ordered logistic regression models were used in sensitivity analyses using the full Likert scale items. For continuous measures (e.g., warmth), linear regression models were used.

Published Protocol ClinicalTrials.gov ID: NCT05127707
