# Supplementary material for: Effect of Exposure to Visual Campaigns and Narrative Vignettes on Addiction Stigma Among Health Care Professionals: A Randomized Clinical Trial
Source: JAMA Netw Open. 2022 Feb 4;5(2):e2146971. doi: 10.1001/jamanetworkopen.2021.46971 (PMC8817201; doi:10.1001/jamanetworkopen.2021.46971)
Supplement: Supplement 2. — eTable 1. Structure of Randomized Clinical Trial eTable 2. Narrative Vignettes Communicating Words Matter and Medication Treatment Works Message Frames eTable 3. Questions Included in Each Domain eTable 4. Comparison of Characteristics of Survey Participants Across Study Arms eTable 5. Effects of Exposure to the Words Matter and Medication Treatment Works Narrative Vignette and Visual Campaign Combination on Stigma Toward People With Opioid Use Disorder Relative to the Visual Campaign–Only Groups eTable 6. Effects of Exposure to the “Words Matter” Visual Campaign and Vignette Frames on Perceptions of Clinically Appropriate Language Related to Substance Use Relative to the No-Exposure Control Group eTable 7. Effects of Exposure to the Words Matter Narrative Vignette and Visual Campaign Combination on Perceptions of Clinically Appropriate Language Related to Substance Use Relative to the Visual Campaign–Only Group eTable 8. Effects of Exposure to the “Medication Treatment Works” Visual Campaign and Vignettes on Stigma toward People with Opioid Use Disorder Receiving Medication Treatment Relative to the No-Exposure Control Group eTable 9. Effects of Exposure to the Words Matter and Medication Treatment Works Message Frames on Stigma Toward People With Opioid Use Disorder Relative to the Nonexposed Control Group Estimated With Ordinal Logistic Regression Models eTable 10. Effects of Exposure to the Words Matter Narrative Vignette and Visual Campaign Combination on Perceptions of Clinically Appropriate Language Related to Substance Use Relative to the Visual Campaign–Only Group Estimated With Ordinal Logistic Regression Models eTable 11. Effects of Exposure to the Medication Treatment Works Visual Campaign and Narrative Vignettes on Stigma Toward People With Opioid Use Disorder Receiving Medication Treatment Relative to the Nonexposed Control Group Estimated With Ordinal Logistic Regression Models eFigure. Words Matter and Medication Treatment Works Visual Campaigns [file jamanetwopen-e2146971-s002.pdf]

Supplementary Online Content

Kennedy-Hendricks A, McGinty EE, Summers A, Krenn S, Fingerhood MI, Barry CL. Effect of exposure to visual campaigns and narrative vignettes on addiction stigma among health care professionals: a randomized clinical trial. *JAMA Netw Open*. 2022;5(2):e2146971. doi:10.1001/jamanetworkopen.2021.46971

eTable 1. Structure of Randomized Clinical Trial

eTable 2. Narrative Vignettes Communicating *Words Matter* and *Medication Treatment Works* Message Frames

eTable 3. Questions Included in Each Domain

eTable 4. Comparison of Characteristics of Survey Participants Across Study Arms

eTable 5. Effects of Exposure to the *Words Matter* and *Medication Treatment Works* Narrative Vignette and Visual Campaign Combination on Stigma Toward People With Opioid Use Disorder Relative to the Visual Campaign–Only Groups

eTable 6. Effects of Exposure to the “Words Matter” Visual Campaign and Vignette Frames on Perceptions of Clinically Appropriate Language Related to Substance Use Relative to the No-Exposure Control Group

eTable 7. Effects of Exposure to the *Words Matter* Narrative Vignette and Visual Campaign Combination on Perceptions of Clinically Appropriate Language Related to Substance Use Relative to the Visual Campaign–Only Group

eTable 8. Effects of Exposure to the “Medication Treatment Works” Visual Campaign and Vignettes on Stigma toward People with Opioid Use Disorder Receiving Medication Treatment Relative to the No-Exposure Control Group

eTable 9. Effects of Exposure to the *Words Matter* and *Medication Treatment Works* Message Frames on Stigma Toward People With Opioid Use Disorder Relative to the Nonexposed Control Group Estimated With Ordinal Logistic Regression Models

eTable 10. Effects of Exposure to the *Words Matter* Narrative Vignette and Visual Campaign Combination on Perceptions of Clinically Appropriate Language Related to Substance Use Relative to the Visual Campaign–Only Group Estimated With Ordinal Logistic Regression Models

eTable 11. Effects of Exposure to the *Medication Treatment Works* Visual Campaign and Narrative Vignettes on Stigma Toward People With Opioid Use Disorder Receiving Medication Treatment Relative to the Nonexposed Control Group Estimated With Ordinal Logistic Regression Models

eFigure. *Words Matter* and *Medication Treatment Works* Visual Campaigns

This supplementary material has been provided by the authors to give readers additional information about their work.

© 2022 Kennedy-Hendricks A et al. *JAMA Network Open*.

eTable 1. Structure of Randomized Clinical Trial

Group	Exposure
1. Control Group	No exposure. Participants proceeded directly to responding to survey questions.
2. Words Matter Visual Campaign	Exposure to visual campaign communicating message frame "Words Matter"
3. Words Matter Visual Campaign + Patient with OUD Vignette	Exposure to visual campaign communicating message frame "Words Matter" followed by exposure to text-based vignette describing importance of non-stigmatizing language from perspective of patient with OUD
4. Words Matter Visual Campaign + Clinician Vignette	Exposure to visual campaign communicating message frame "Words Matter" followed by exposure to text-based vignette describing importance of non-stigmatizing language from perspective of clinician
5. Words Matter Visual Campaign + Health System Administrator Vignette	Exposure to visual campaign communicating message frame "Words Matter" followed by exposure to text-based vignette describing importance of non-stigmatizing language from perspective of health system administrator
6. Medication Treatment Works Visual Campaign	Exposure to visual campaign communicating message frame "Medication Treatment Works"
7. Medication Treatment Works Visual Campaign + Patient with OUD Vignette	Exposure to visual campaign communicating message frame "Medication Treatment Works" followed by text-based vignette describing value of medication treatment from perspective of patient with OUD treated with medication
8. Medication Treatment Works Visual Campaign + Clinician Vignette	Exposure to visual campaign communicating message frame "Medication Treatment Works" followed by text-based vignette describing value of medication treatment from perspective of clinician
9. Medication Treatment Works Visual Campaign + Health System Administrator Vignette	Exposure to visual campaign communicating message frame "Medication Treatment Works" followed by text-based vignette describing value of medication treatment from perspective of health administrator

© 2022 Kennedy-Hendricks A et al. *JAMA Network Open*.

eTable 2. Narrative Vignettes Communicating *Words Matter* and *Medication Treatment Works* Message Frames

Message Frame: Words Matter			Message Frame: Medication Treatment for OUD Works		
**Patient (words=165)**	**Provider (words=156)**	**Administrator (words=164)**	**Patient (words=163)**	**Provider (words=166)**	**Administrator (words=178)**
For the past 5 years, I have been working to overcome my opioid use disorder.<br><br>I used to dread telling clinicians about my diagnosis of opioid use disorder. They used language like "addict" and "drug abuser" when they spoke to me and about me. They would regularly ask me if I was "clean."<br><br>When they used these words, it felt like they didn't see me as a person. That all they could see was my addiction.<br>Last year, things started to change.<br><br>I was in the health center and had a great clinician who spoke to me like a human	For the past 5 years, an increasing number of my patients have had opioid use disorder.<br><br>I used to refer to my patients with opioid use disorder using language like "addict" and "drug abuser." I would regularly ask if they were "clean."<br><br>But, after talking with an addiction medicine colleague, I realized that this language made my patients feel like I didn't see them as people. That all I could see was their addiction. Last year, things started to change.<br><br>I began being more intentional with my patients about the language I used. I even began asking my colleagues to refrain	For the past 5 years, I have been the CEO of a health system with an increasing number of patients with opioid use disorder.<br><br>In our health system, our clinical staff used to refer to patients with opioid use disorder using language like "addict" or "drug abuser." These patients were regularly asked if they were "clean."<br><br>But, after talking with an addiction medicine colleague, I realized that this language made our patients feel like we didn't see them as people. That all we could see was their addiction.<br>Last year, things started to change.	For the past 5 years, I have been working to overcome my opioid use disorder.<br><br>I had tried to stop using opioids many times. Typically, I would be referred to counseling, but then have a really hard time managing cravings and the pain of withdrawal.<br><br>For me, counseling alone didn't work, and I always returned to using opioids.<br><br>These experiences left me feeling discouraged and wondering whether I would ever recover.<br><br>Last year, things started to change.	For the past 5 years, an increasing number of my patients have had opioid use disorder.<br><br>I have seen patients who have tried to stop using opioids many times. Typically, I would refer them to counseling, but these patients had a really hard time managing cravings and the pain of withdrawal.<br><br>For most, counseling didn't work, and they always returned to using opioids.<br><br>These experiences left me feeling discouraged and wondering whether these patients would ever recover.	For the past 5 years, I have been the CEO of a health system with an increasing number of patients with opioid use disorder.<br><br>In our system, we saw patients who had tried to stop using opioids many times. Typically, we would refer them to counseling, but these patients had a really hard time managing cravings and the pain of withdrawal.<br><br>For most, counseling didn't work, and they always returned to using opioids.<br><br>These experiences left our clinicians feeling discouraged and wondering whether these patients would ever recover.

© 2022 Kennedy-Hendricks A et al. *JAMA Network Open*.

being. I even heard her asking one of the other clinicians not to call me an “addict” because she said it was not respectful.<br><br>It may seem like a little thing, but it made me feel like I belonged there, and that people cared about me.<br><br>Health care professionals can be role models and what they say and do makes a big difference to me.	from using terms like addict” because I realized that it is just not respectful.<br><br>It may seem like a little thing, but I think it is part of communicating to my patients that I care.<br><br>Health care professionals can be role models and what we say and do makes a big difference in people’s lives.	I began instituting policies encouraging our health system staff to use more clinically appropriate language and refrain from using terms like “addict” because it is just not respectful.<br><br>It may seem like a little thing, but I think it is part of us communicating to our patients that we care.<br><br>Health care professionals can be role models and what we say and do makes a big difference in people’s lives.	I had a great clinician who suggested that I consider trying an FDA-approved medication to treat opioid use disorder. She told me these medications are very effective.<br><br>My experience using medication to treat my opioid use disorder gave me hope that I could manage withdrawal and get on the path to recovery.<br><br>I am proud to say that I am now in recovery from opioid use disorder.<br><br>Health care professionals can be role models and what they say and do makes a big difference to me.	Last year, things started to change.<br><br>I learned that FDA-approved medications to treat opioid use disorder are very effective and began encouraging my patients to consider them.<br><br>My experiences treating patients’ opioid use disorder with medication gave me hope that they could manage withdrawal and get on the path to recovery.<br><br>I am proud to say that many of my patients are now in recovery from opioid use disorder.<br><br>Health care professionals can be role models and what we say and do makes a big difference in people’s lives.	Last year, things started to change.<br><br>We began teaching clinicians that FDA-approved medications to treat opioid use disorder are very effective, and encouraging our patients consider them.<br><br>Our system’s experiences treating patients’ opioid use disorder with medication gave us hope that they could manage withdrawal and get on the path to recovery.<br><br>I am proud to say that many of our patients are now in recovery from opioid use disorder.<br><br>Health care professionals can be role models and what we say and do makes a big difference in people’s lives.

© 2022 Kennedy-Hendricks A et al. *JAMA Network Open*.

eTable 3. Questions Included in Each Domain

Survey Question	Response Options
Domain A	
How willing would you be to have a person with opioid use disorder marry into your family?	5-point Likert scale ranging from strongly willing to strongly unwilling
How willing would you be to have a person with opioid use disorder as a neighbor?	5-point Likert scale ranging from strongly willing to strongly unwilling
Indicate the extent to which you agree or disagree with the following statement: Opioid use disorder is a chronic medical condition like diabetes mellitus.	5-point Likert scale ranging from strongly agree to strongly disagree
Indicate the extent to which you agree or disagree with the following statement: Individuals with opioid use disorder only have themselves to blame for their problem.	5-point Likert scale ranging from strongly agree to strongly disagree
Do you favor or oppose increasing government spending on treatment of opioid use disorder?	5-point Likert scale ranging from strongly oppose to strongly favor
On a scale from 0-100, where 0 = extremely cold, 50 = neutral, and 100 = extremely warm, how warmly (or coldly) do you feel toward people with opioid use disorder?	0-100 scale
Domain B	
Do you agree or disagree that using the term "addict" is appropriate in a clinical care setting?	5-point Likert scale ranging from strongly agree to strongly disagree
Do you agree or disagree that using the term "substance abuse" is appropriate in a clinical care setting?	5-point Likert scale ranging from strongly agree to strongly disagree
Do you agree or disagree that using the term "dirty" in reference to result from drug test is appropriate in a clinical care setting?	5-point Likert scale ranging from strongly agree to strongly disagree
Do you agree or disagree that using the term "clean" in reference to result from drug test is appropriate in a clinical care setting?	5-point Likert scale ranging from strongly agree to strongly disagree

© 2022 Kennedy-Hendricks A et al. *JAMA Network Open*.

Do you agree or disagree that using the term "addicted baby" is appropriate in a clinical care setting?	5-point Likert scale ranging from strongly agree to strongly disagree
Do you agree or disagree that using the term "person with substance use disorder" is appropriate in a clinical care setting?	5-point Likert scale ranging from strongly agree to strongly disagree
Do you agree or disagree that using the term "substance use" is appropriate in a clinical care setting?	5-point Likert scale ranging from strongly agree to strongly disagree
Do you agree or disagree that using the term "negative" in reference to result from drug test is appropriate in a clinical care setting?	5-point Likert scale ranging from strongly agree to strongly disagree
Do you agree or disagree that using the term "positive" in reference to result from drug test is appropriate in a clinical care setting?	5-point Likert scale ranging from strongly agree to strongly disagree
Do you agree or disagree that using the term "baby born with neonatal opioid withdrawal syndrome" is appropriate in a clinical care setting?	5-point Likert scale ranging from strongly agree to strongly disagree
Signing a "Words Matter" pledge means that you are committing to using non-stigmatizing language about substance use disorder when working in clinical settings. Would you be willing to sign a "Words Matter" pledge?	Yes, No
Domain C	
To the best of your knowledge, is there a treatment for opioid use disorder that is effective for a long period of time, or isn't there such a treatment?	Yes, there is such a treatment.<br>No, there isn't such a treatment.<br>Don't know.
How willing would you be to have a person taking medication to treat opioid use disorder marry into your family?	5-point Likert scale ranging from strongly willing to strongly unwilling
How willing would you be to have a person taking medication to treat opioid use disorder as a neighbor?	5-point Likert scale ranging from strongly willing to strongly unwilling
Using the scale below, do you think that a person taking medication to treat opioid use disorder is:	5-point Likert scale ranging from "weak" to "strong"

© 2022 Kennedy-Hendricks A et al. *JAMA Network Open*.

Indicate the extent to which you agree or disagree with the following statement: Medication treatment for opioid use disorder is more effective than treatment without medication.	5-point Likert scale ranging from strongly agree to strongly disagree
Indicate the extent to which you agree or disagree with the following statement: Most people with opioid use disorder will, with medication treatment, get well and return to productive lives.	5-point Likert scale ranging from strongly agree to strongly disagree
On a scale from 0-100, where 0 = extremely cold, 50 = neutral, and 100 = extremely warm, how warmly (or coldly) do you feel toward people taking medication to treat opioid use disorder?	0-100 scale

© 2022 Kennedy-Hendricks A et al. *JAMA Network Open*.

eTable 4. Comparison of Characteristics of Survey Participants Across Study Arms (N = 1842)

		Words Matter Frame				Medication Works Frame				
	No-Exposure Control	Poster	Poster + Patient Vignette	Poster + Clinician Vignette	Poster + Hospital Admin Vignette	Poster	Poster + Patient Vignette	Poster + Clinician Vignette	Poster + Hospital Admin Vignette	*p-value*
	N=202	N=206	N=203	N=205	N=205	N=205	N=205	N=204	N=208	
Age, mean (SD)	46.7 (13.3)	46.5(12.0)	47.3 (13.3)	46.6 (12.3)	46.3(13.0)	46.4 (13.0)	47.5 (12.8)	47.7 (12.6)	47.8 (13.0)	0.88
Education, n (%)										
High School or Less	9 (4.5%)	12 (5.8%)	7 (3.4%)	15 (7.3%)	5 (2.4%)	9 (4.4%)	13 (6.3%)	9 (4.4%)	8 (3.8%)	0.63
Some College	49 (24.4%)	44 (21.4%)	55 (27.1%)	52 (25.4%)	45 (22.0%)	53 (25.9%)	43 (21.0%)	47 (23.0%)	43 (20.7%)	
Bachelor's or Greater	143 (71.1%)	150 (72.8%)	141 (69.5%)	138 (67.3%)	155 (75.6%)	143 (69.8%)	149 (72.7%)	148 (72.5%)	157 (75.5%)	
Female, n (%)	133 (66.2%)	143 (69.4%)	150 (73.9%)	152 (74.1%)	140 (68.3%)	148 (72.2%)	163 (79.5%)	147 (72.1%)	146 (70.2%)	0.14
Prescribing clinician, n (%)	64 (31.8%)	72 (35.0%)	69 (34.0%)	61 (29.8%)	68 (33.2%)	57 (27.8%)	54 (26.3%)	68 (33.3%)	70 (33.7%)	0.55
White, Not-Hispanic, n (%)	151 (75.1%)	140 (68.0%)	149 (73.4%)	148 (72.2%)	143 (69.8%)	162 (79.0%)	151 (73.7%)	142 (69.6%)	158 (76.0%)	0.25
Household Income, n (%)										
<$50,000	31 (15.4%)	24 (11.7%)	29 (14.3%)	29 (14.1%)	34 (16.6%)	26 (12.7%)	41 (20.0%)	26 (12.7%)	21 (10.1%)	0.86
$50,000-$99,999	51 (25.4%)	67 (32.5%)	69 (34.0%)	64 (31.2%)	58 (28.3%)	60 (29.3%)	65 (31.7%)	62 (30.4%)	64 (30.8%)	
$100,000-$149,999	41 (20.4%)	44 (21.4%)	39 (19.2%)	45 (22.0%)	48 (23.4%)	53 (25.9%)	42 (20.5%)	48 (23.5%)	45 (21.6%)	
$150,000-$199,999	26 (12.9%)	26 (12.6%)	25 (12.3%)	25 (12.2%)	20 (9.8%)	24 (11.7%)	22 (10.7%)	21 (10.3%)	26 (12.5%)	
>$200,000	52 (25.9%)	45 (21.8%)	41 (20.2%)	42 (20.5%)	45 (22.0%)	42 (20.5%)	35 (17.1%)	47 (23.0%)	52 (25.0%)	
Married or Living with Partner, n (%)	134 (66.7%)	145 (70.4%)	145 (71.4%)	142 (69.3%)	143 (69.8%)	155 (75.6%)	135 (65.9%)	140 (68.6%)	146 (70.2%)	0.63

© 2022 Kennedy-Hendricks A et al. *JAMA Network Open*.

Living in Single-Family House, n (%)	170 (84.6%)	178 (86.4%)	175 (86.2%)	179 (87.3%)	177 (86.3%)	171 (83.4%)	177 (86.3%)	181 (88.7%)	186 (89.4%)	0.77
Household Size, n (%)										
1	44 (21.9%)	42 (20.4%)	36 (17.7%)	35 (17.1%)	38 (18.5%)	31 (15.1%)	43 (21.0%)	35 (17.2%)	45 (21.6%)	0.98
2	60 (29.9%)	70 (34.0%)	71 (35.0%)	65 (31.7%)	69 (33.7%)	70 (34.1%)	77 (37.6%)	73 (35.8%)	66 (31.7%)	
3	35 (17.4%)	37 (18.0%)	31 (15.3%)	39 (19.0%)	36 (17.6%)	40 (19.5%)	31 (15.1%)	38 (18.6%)	36 (17.3%)	
4 or more	62 (30.8%)	57 (27.7%)	65 (32.0%)	66 (32.2%)	62 (30.2%)	64 (31.2%)	54 (26.3%)	58 (28.4%)	61 (29.3%)	
Own home, n (%)	162 (80.6%)	166 (80.6%)	163 (80.3%)	174 (84.9%)	173 (84.4%)	156 (76.1%)	159 (77.6%)	175 (85.8%)	177 (85.1%)	0.09
Household Head, n (%)	173 (86.1%)	188 (91.3%)	174 (85.7%)	184 (89.8%)	189 (92.2%)	190 (92.7%)	184 (89.8%)	182 (89.2%)	191 (91.8%)	0.18
Metropolitan Area, n (%)	172 (85.6%)	180 (87.4%)	177 (87.2%)	182 (88.8%)	182 (88.8%)	182 (88.8%)	178 (86.8%)	182 (89.2%)	187 (89.9%)	0.94
Region of US, n (%)										
Northeast	37 (18.4%)	43 (20.9%)	45 (22.2%)	48 (23.4%)	32 (15.6%)	37 (18.0%)	36 (17.6%)	29 (14.2%)	38 (18.3%)	0.12
Midwest	54 (26.9%)	54 (26.2%)	56 (27.6%)	51 (24.9%)	56 (27.3%)	60 (29.3%)	52 (25.4%)	59 (28.9%)	70 (33.7%)	
South	58 (28.9%)	76 (36.9%)	63 (31.0%)	74 (36.1%)	81 (39.5%)	72 (35.1%)	75 (36.6%)	84 (41.2%)	56 (26.9%)	
West	52 (25.9%)	33 (16.0%)	39 (19.2%)	32 (15.6%)	36 (17.6%)	36 (17.6%)	42 (20.5%)	32 (15.7%)	44 (21.2%)	
Health Profession, n (%)										
Physician	56 (27.9%)	57 (27.7%)	49 (24.1%)	48 (23.4%)	54 (26.3%)	45 (22.0%)	43 (21.0%)	59 (28.9%)	56 (26.9%)	0.15
NP or PA	8 (4.0%)	15 (7.3%)	20 (9.9%)	13 (6.3%)	14 (6.8%)	12 (5.9%)	11 (5.4%)	9 (4.4%)	14 (6.7%)	
RN or LPN	52 (25.9%)	62 (30.1%)	44 (21.7%)	52 (25.4%)	54 (26.3%)	71 (34.6%)	51 (24.9%)	64 (31.4%)	55 (26.4%)	
Therapist	10 (5.0%)	13 (6.3%)	11 (5.4%)	16 (7.8%)	14 (6.8%)	7 (3.4%)	11 (5.4%)	13 (6.4%)	11 (5.3%)	
Health Technician or Technologist	20 (10.0%)	14 (6.8%)	16 (7.9%)	23 (11.2%)	18 (8.8%)	25 (12.2%)	29 (14.1%)	17 (8.3%)	29 (13.9%)	
Health Aid or Assistant	31 (15.4%)	26 (12.6%)	27 (13.3%)	22 (10.7%)	28 (13.7%)	23 (11.2%)	23 (11.2%)	16 (7.8%)	23 (11.1%)	
Other Practitioner	24 (11.9%)	19 (9.2%)	36 (17.7%)	31 (15.1%)	23 (11.2%)	22 (10.7%)	37 (18.0%)	26 (12.7%)	20 (9.6%)	

© 2022 Kennedy-Hendricks A et al. *JAMA Network Open*.

eTable 5. Effects of Exposure to the *Words Matter* and *Medication Treatment Works* Narrative Vignette and Visual Campaign Combination on Stigma Toward People With Opioid Use Disorder Relative to the Visual Campaign–Only Groups

	Percentage Point Differences in Attitudes Between Vignette Exposure and Poster Groups (95% CI) *p-value*							
	Words Matter				Medication Treatment Works			
	Visual Campaign Only	Visual Campaign + Patient Vignette	Visual Campaign + Clinician Vignette	Visual Campaign + Administrator Vignette	Visual Campaign Only	Visual Campaign + Patient Vignette	Visual Campaign + Clinician Vignette	Visual Campaign + Administrator Vignette
Unwilling to have person with OUD marry into family	70.9%	-17.6 (-26.8, -8.4) <0.001	-10.9 (-20.0, -1.8) 0.018	-6.8 (-15.8, 2.1) 0.133	64.9%	-9.0 (-18.3, 0.4) 0.061	-1.9 (-11.2, 7.4) 0.692	-3.6 (-12.8, 5.7) 0.447
Unwilling to have person with OUD as neighbor	44.7%	-12.1 (-21.5, -2.8) 0.011	-6.0 (-15.5, 3.5) 0.218	-7.5 (-17.0, 2.0) 0.120	39.0%	-9.9 (-19.0, -0.7) 0.035	-3.4 (-12.7, 6.0) 0.478	-4.4 (-13.6, 4.8) 0.344
Agree OUD is a medical condition	60.7%	0.1 (-9.2, 9.4) 0.985	-0.6 (-10.0, 8.8) 0.895	3.2 (-6.0, 12.4) 0.498	56.1%	3.0 (-6.3, 12.3) 0.523	1.4 (-7.9, 10.6) 0.770	3.9 (-5.3, 13.1) 0.404
Agree people with OUD not to blame	51.0%	6.1 (-3.5, 15.8) 0.213	1.9 (-7.8, 11.6) 0.700	9.6 (0.1, 19.0) 0.048	52.7%	5.0 (-4.6, 14.5) 0.308	5.6 (-3.9, 15.1) 0.247	1.8 (-7.7, 11.2) 0.715
Favor increased spending on OUD treatment	62.6%	9.2 (0.1, 18.3) 0.047	1.5 (-7.8, 10.8) 0.755	4.8 (-4.4, 14.0) 0.304	62.0%	3.6 (-5.6, 12.8) 0.440	6.4 (-2.8, 15.6) 0.171	4.6 (-4.6, 13.8) 0.323
	Difference in Warmth (on Scale of 0-100) Between Vignette Exposure and Poster Groups (95% CI)							
	Words Matter				Medication Treatment Works			
	Visual Campaign Only	Visual Campaign + Patient Vignette	Visual Campaign + Clinician Vignette	Visual Campaign + Administrator Vignette	Visual Campaign Only	Visual Campaign + Patient Vignette	Visual Campaign + Clinician Vignette	Visual Campaign + Administrator Vignette

© 2022 Kennedy-Hendricks A et al. *JAMA Network Open*.

Degree of warmth toward people with OUD	50.5	7.6 (3.3, 11.8) 0.001	1.0 (-3.3, 5.3) 0.636	5.9 (1.5, 10.2) 0.008	53.7	7.6 (3.3, 11.8) <0.001	1.0 (-3.3, 5.3) 0.636	5.9 (1.5, 10.2) 0.008

Logit regression models estimated differences between vignette exposure and poster-only groups. Percentage point differences and differences in warmth estimated through post-estimation marginal effects. Model estimates adjust for age (continuous), female gender, prescriber status (physician, nurse practitioner, or physician assistant), and white and not Hispanic or other race or Hispanic ethnicity.

© 2022 Kennedy-Hendricks A et al. *JAMA Network Open*.

eTable 6. Effects of Exposure to the "Words Matter" Visual Campaign and Vignette Frames on Perceptions of Clinically Appropriate Language Related to Substance Use Relative to the No-Exposure Control Group

	**No-Exposure Control Group**	**Visual Campaign Only**	**Visual Campaign + Patient Vignette**	**Visual Campaign + Clinician Vignette**	**Visual Campaign + Administrator Vignette**
**Stigmatizing Terms**	% Agree Appropriate	Percentage Point Differences in Endorsement of Language as Appropriate Between Exposure and No-Exposure Control Group (95% CI) *p-value*			
Addict	46.3%	-4.7 (-14.3, 4.9) *0.340*	-23.1 [a] (-32.1, -14.2) *<0.001*	-16.4 [a] (-25.6, -7.1) *0.001*	-20.4 [a] (-29.5, -11.3) *<0.001*
Substance Abuse	81.1%	-7.3 (-15.4, 0.8) *0.078*	-23.3 [a] (-31.9, -15.0) *<0.001*	-18.2 [a] (-26.7, -9.6) *<0.001*	-17.5 [a] (-26.0, -9.1) *<0.001*
Dirty in Reference to Result from Drug Test	18.4%	-5.5 (-12.5, 1.5) *0.125*	-9.0 [a] (-15.7, -2.3) *0.009*	-7.9 [a] (-14.7, -1.2) *0.021*	-9.6 [a] (-16.1, -3.0) *0.004*
Clean in Reference to Result from Drug Test	54.2%	-12.7 [a] (-22.4, -3.1) *0.010*	-22.9 [a] (-32.2, -13.5) *<0.001*	-16.2 [a] (-25.8, -6.6) *0.001*	-25.3 [a] (-34.6, -16.1) *<0.001*
Addicted Baby	31.3%	-4.8 (-13.4, 3.7) *0.270*	-14.3 (-22.3, -6.3) *<0.001*	-12.1 [a] (-20.2, -4.0) *0.003*	-14.8 [a] (-22.7, -7.0) *<0.001*
	Scale Mean	Change on 5-point Scale (95% CI) *p-value*			
Endorsement of Stigmatizing Terms (Range 1-5)	3.1	-0.2 [a] (-0.3, -0.0) *0.027*	-0.5 [a] (-0.7, -0.4) *<0.001*	-0.4 [a] (-0.5, -0.2) *<0.001*	-0.5 [a] (-0.7, -0.4) *<0.001*
**Non-Stigmatizing Alternatives**	% Agree Appropriate	Percentage Point Differences in Endorsement of Language as Appropriate Between Exposure and No-Exposure Control Group			

© 2022 Kennedy-Hendricks A et al. *JAMA Network Open*.

		(95% CI)<br>*p-value*			
Person with Substance Use Disorder	80.1%	1.5<br>(-6.2, 9.2)<br>*0.704*	1.1<br>(-6.6, 8.8)<br>*0.781*	1.5<br>(-6.2, 9.2)<br>*0.697*	1.9<br>(-5.7, 9.6)<br>*0.618*
Substance Use	85.6%	-1.2<br>(-8.1, 5.8)<br>*0.745*	-3.5<br>(-10.7, 3.7)<br>*0.347*	-4.0<br>(11.2, 3.2)<br>*0.277*	-2.6<br>(-9.7, 4.5)<br>*0.469*
Negative in Reference to Result from Drug Test	77.1%	1.3<br>(-6.9, 9.4)<br>*0.756*	-5.0<br>(-13.6, 3.5)<br>*0.245*	-2.7<br>(-11.0, 5.6)<br>*0.529*	-2.5<br>(-10.9, 5.9)<br>*0.554*
Positive in Reference to Result from Drug Test	81.1%	2.9<br>(-4.6, 10.3)<br>*0.448*	-4.0<br>(-11.8, 3.9)<br>*0.323*	-7.3<br>(-15.3, 0.8)<br>*0.076*	-6.7<br>(-14.7, 1.4)<br>*0.104*
Baby Born with Neonatal Opioid Withdrawal Syndrome	88.6%	-3.6<br>(-10.1, 3.0)<br>*0.288*	-9.1 [a]<br>(-16.2, -2.0)<br>*0.012*	-7.5 [a]<br>(-14.4, -0.6)<br>*0.033*	-7.4 [a]<br>(-14.3, -0.5)<br>0.035
	Scale Mean	Change on 5-point Scale<br>(95% CI)<br>*p-value*			
Endorsement of Non-Stigmatizing Alternatives (Range 1-5)	4.3	-0.0<br>(-0.2, 0.1)<br>*0.633*	-0.1<br>(-0.2, 0.0)<br>*0.092*	-0.1<br>(-0.2, 0.0)<br>*0.100*	-0.2 [a]<br>(-0.3, -0.0)<br>*0.014*
**Language Pledge**		Percentage Point Differences in Willingness to Sign Pledge Between Exposure and No-Exposure Control Group<br>(95% CI)<br>*p-value*			
Willing to Sign Pledge to Use Non-Stigmatizing Language	74.4%	-3.0<br>(-15.4, 9.4)<br>*0.633*	0.4<br>(-7.9, 8.8)<br>*0.917*	-1.6<br>(-23.0, 2.0)<br>*0.100*	3.4<br>(-4.7, 11.6)<br>*0.407*

[a] Indicates sharpened False Discovery Rate q-value<0.05.
Logit regression models estimated differences between exposure and no-exposure control groups. Percentage point differences estimated through post-estimation marginal effects. Model estimates adjust for age (continuous), female gender, prescriber status (physician, nurse

© 2022 Kennedy-Hendricks A et al. *JAMA Network Open*.

practitioner, or physician assistant), and white and not Hispanic or other race or Hispanic ethnicity. N=1,020 (excludes participants randomized to Medication Treatment Works message frames who were not asked questions regarding stigmatizing terms).

© 2022 Kennedy-Hendricks A et al. *JAMA Network Open*.

eTable 7. Effects of Exposure to the *Words Matter* Narrative Vignette and Visual Campaign Combination on Perceptions of Clinically Appropriate Language Related to Substance Use Relative to the Visual Campaign–Only Group

Language appropriate for use in clinical setting	Percentage Point Differences in Endorsement of Language as Appropriate in Clinical Setting Between Vignette Exposure and Poster Groups (95% CI) p-value			
	Poster Only	Patient Vignette	Clinician Vignette	Administrator Vignette
Stigmatizing Terms				
Addict	40.8%	-18.5<br>(-27.3, -9.7)<br><0.001	-11.8<br>(-20.9, -2.6)<br>0.012	-15.7<br>(-24.7, -6.8)<br><0.001
Substance Abuse	72.8%	-16.0<br>(-25.0, -6.9)<br><0.001	-10.8<br>(-20.0, -1.9)<br>0.017	-10.2<br>(-19.0, -1.3)<br>0.024
Dirty in Reference to Result from Drug Test	12.6%	-3.2<br>(-9.3, 2.8)<br>0.295	-2.3<br>(-8.4, 3.7)<br>0.452	-4.2<br>(-10.0, 1.6)<br>0.155
Clean in Reference to Result from Drug Test	41.3%	-10.1<br>(-19.3, -0.9)<br>0.032	-3.5<br>(-13.0, 5.9)<br>0.465	-12.6<br>(-21.8, -3.4)<br>0.007
Addicted Baby	25.2%	-9.3<br>(-17.1, -1.4)<br>0.020	-7.1<br>(-15.0, 0.8)<br>0.077	-10.0<br>(-17.7, -2.2)<br>0.012
Endorsement of Stigmatizing Terms Scale (Range 1-5)	2.9	-0.4<br>(-0.5, -0.2)<br><0.001	-21.0<br>(-36.7, -5.2)<br>0.009	-34.8<br>(-50.2, -19.4)<br><0.001
Non-Stigmatizing Alternatives				
Person with Substance Use Disorder	81.6%	-0.5<br>(-8.1, 7.0)<br>0.889	-0.1<br>(-7.7, 7.4)<br>0.978	0.5<br>(-6.9, 7.9)<br>0.893
Substance Use	84.5%	-2.3<br>(-9.5, 4.9)<br>0.535	-2.8<br>(-10.0, 4.4)<br>0.444	-1.4<br>(-8.5, 5.7)<br>0.701
Negative in Reference to Result from Drug Test	77.7%	-6.4<br>(-14.7, 2.0)<br>0.134	-4.1<br>(-12.2, 4.1)<br>0.330	-3.8<br>(-12.0, 4.4)<br>0.362
Positive in Reference to Result from Drug Test	83.5%	-6.7<br>(-14.4, 1.0)<br>0.088	-10.9<br>(-17.9, -2.3)<br>0.012	-9.6<br>(-17.4, -1.8)<br>0.016
Baby Born with Neonatal Opioid Withdrawal Syndrome	85.0%	-5.5<br>(-13.0, 1.9)<br>0.143	-3.9<br>(-11.1, 3.4)<br>0.294	-3.7<br>(-10.9, 3.4)<br>0.305

© 2022 Kennedy-Hendricks A et al. *JAMA Network Open*.

Endorsement of Less Stigmatizing Terms Scale (Range 1-5)	4.2	-7.6<br>(-19.4, 4.3)<br>0.212	-7.6<br>(-20.0, 4.8)<br>0.230	-12.0<br>(-23.8, -0.3)<br>0.045
Willing to Sign Pledge to Use Non-Stigmatizing Language	70.9%	5.0<br>(-3.5, 13.6)<br>0.247	2.9<br>(-5.7, 11.6)<br>0.503	8.1<br>(-0.2, 16.4)<br>0.055

Logit regression models estimated differences between exposure and no-exposure control groups. Percentage point differences estimated through post-estimation marginal effects. Model estimates adjust for age (continuous), female gender, prescriber status (physician, nurse practitioner, or physician assistant), and white and not Hispanic or other race or Hispanic ethnicity.

© 2022 Kennedy-Hendricks A et al. *JAMA Network Open*.

eTable 8. Effects of Exposure to the "Medication Treatment Works" Visual Campaign and Vignettes on Stigma toward People with Opioid Use Disorder Receiving Medication Treatment Relative to the No-Exposure Control Group

	No-Exposure Control Group	Percentage Point Differences in Attitudes Between Exposure and No-Exposure Control Groups (95% CI) *p-value*			
		Visual Campaign Only	Visual Campaign + Patient Vignette	Visual Campaign + Clinician Vignette	Visual Campaign + Administrator Vignette
There is a treatment for OUD that is effective for a long period of time.	53.7%	0.1<br>(-9.2, 9.5)<br>*0.978*	-0.4<br>(-9.8, 9.1)<br>*0.939*	4.8<br>(-4.6, 14.2)<br>*0.317*	-0.5<br>(-9.9, 9.0)<br>*0.926*
Unwilling to have a person taking medication to treat OUD marry into family	41.3%	-2.0<br>(-11.5, 7.5)<br>*0.675*	-2.4<br>(-11.8, 7.1)<br>*0.625*	-3.5<br>(-12.9, 5.8)<br>*0.459*	-4.0<br>(-13.3, 5.2)<br>*0.392*
Unwilling to have a person taking medication to treat OUD as neighbor	21.4%	-2.0<br>(-9.7, 5.8)<br>*0.621*	-5.3<br>(-12.8, 2.3)<br>*0.172*	-1.6<br>(-9.4, 6.2)<br>*0.681*	-0.3<br>(-8.0, 7.5)<br>*0.944*
Person taking medication with OUD is strong	55.7%	1.0<br>(-8.6, 10.6)<br>*0.839*	4.0<br>(-5.6, 13.6)<br>*0.417*	5.8<br>(-3.7, 15.3)<br>*0.232*	1.0<br>(-8.5, 10.6)<br>*0.834*
Agree MOUD is more effective than treatment without medication	74.6%	-6.5<br>(-15.0, 2.0)<br>*0.132*	-4.5<br>(-12.9, 4.0)<br>*0.299*	-8.1<br>(-16.8, 0.6)<br>*0.069*	-8.3<br>(-16.9, 0.3)<br>*0.057*
Most people with OUD will, with medication treatment, get well and return to productive lives	48.8%	5.9<br>(-3.8, 15.5)<br>*0.236*	3.8<br>(-6.0, 13.5)<br>*0.447*	-0.1<br>(-9.8, 9.6)<br>*0.979*	-0.4<br>(-10.0, 9.3)<br>*0.943*
	No-Exposure Control Group	Difference in Warmth (on Scale of 0-100) Between Exposure and No-Exposure Control Groups (95% CI) *p-value*			
		Visual Campaign Only	Visual Campaign + Patient Vignette	Visual Campaign + Clinician Vignette	Visual Campaign + Administrator Vignette
Degree of warmth toward people taking	66.1	0.6<br>(-3.7, 4.9)<br>*0.793*	4.6[a]<br>(0.3, 9.0)<br>*0.037*	1.0<br>(-3.1, 5.1)<br>*0.627*	4.4[a]<br>(0.2, 8.6)<br>*0.041*

© 2022 Kennedy-Hendricks A et al. *JAMA Network Open*.

medication to treat OUD					

[a] Indicates sharpened False Discovery Rate q-value<0.05.
Logit regression models estimated differences between exposure and no-exposure control groups. Percentage point differences and differences in warmth estimated through post-estimation marginal effects. Model estimates adjust for age (continuous), female gender, prescriber status (physician, nurse practitioner, or physician assistant), and white and not Hispanic or other race or Hispanic ethnicity. N=1,023 (excludes participants randomized to Words Matter message frames who were not asked questions regarding stigmatizing terms).

© 2022 Kennedy-Hendricks A et al. *JAMA Network Open*.

eTable 9. Effects of Exposure to the *Words Matter* and *Medication Treatment Works* Message Frames on Stigma Toward People With Opioid Use Disorder Relative to the Nonexposed Control Group Estimated With Ordinal Logistic Regression Models

	Coefficient (95% CI) *p-value*							
	Words Matter Message Frame				Medication Treatment Works Message Frame			
Outcomes	Visual Campaign Only	Visual Campaign + Patient Vignette	Visual Campaign + Clinician Vignette	Visual Campaign + Administrator Vignette	Visual Campaign Only	Visual Campaign + Patient Vignette	Visual Campaign + Clinician Vignette	Visual Campaign + Administrator Vignette
Unwillingness to have person with OUD marry into family	-0.03 (-0.39, 0.32) *0.848*	-0.74 (-1.11, -0.36) *<0.001*	-0.48 (-0.83, -0.13) *0.007*	-0.34 (-0.68, -0.00) *0.050*	-0.34 (-0.68, 0.01) *0.058*	-0.52 (-0.89, -0.15) *0.006*	-0.37 (-0.71, -0.03) *0.035*	-0.38 (-0.72, -0.03) *0.033*
Unwillingness to have person with OUD as neighbor	-0.02 (-0.40, 0.36) *0.918*	-0.77 (-1.15, -0.39) *<0.001*	-0.34 (-0.70, -0.01) *0.058*	-0.36 (-0.71, -0.02) *0.040*	-0.27 (-0.62, 0.08) *0.137*	-0.76 (-1.12, -0.39) *<0.001*	-0.35 (-0.70, 0.00) *0.051*	-0.44 (-0.79, -0.09) *0.013*
Agreement that OUD is a medical condition	-0.13 (-0.45, -0.19) *0.417*	-0.26 (-0.62, 0.10) *0.160*	-0.28 (-0.61, 0.06) *0.107*	-0.34 (-0.66, -0.02) *0.038*	-0.10 (-0.44, 0.24) *0.566*	-0.30 (-0.64, 0.04) *0.084*	-0.07 (-0.39, 0.24) *0.647*	-0.37 (-0.72, -0.02) *0.040*
Agreement people with OUD not to blame	-0.27 (-0.62, 0.08) *0.124*	0.02 (-0.32, 0.36) *0.910*	-0.18 (-0.52, 0.16) *0.311*	0.17 (-0.21, 0.54) *0.379*	-0.22 (-0.56, 0.13) *0.215*	0.10 (-0.25, 0.45) *0.563*	-0.05 (-0.38, 0.27) *0.744*	0.00 (-0.33, 0.33) *0.998*
Support for increased spending on OUD treatment	-0.05 (-0.39, 0.29) *0.766*	0.30 (-0.04, 0.65) 0.082	-0.06 (-0.41, 0.29) *0.745*	0.16 (-0.17, 0.49) *0.341*	-0.01 (-0.36, 0.35) *0.977*	0.21 (-0.15, 0.57) *0.257*	0.20 (-0.15, 0.54) *0.261*	0.18 (-0.17, 0.53) *0.316*

Ordered logistic regression coefficients display average difference on 5-point Likert scale outcome associated with exposure to message frame relative to no-exposure control group. Model estimates adjust for age (continuous), female gender, prescriber status (physician, nurse practitioner, or physician assistant), and white and not Hispanic or other race or Hispanic ethnicity.

© 2022 Kennedy-Hendricks A et al. *JAMA Network Open*.

eTable 10. Effects of Exposure to the *Words Matter* Narrative Vignette and Visual Campaign Combination on Perceptions of Clinically Appropriate Language Related to Substance Use Relative to the Visual Campaign–Only Group Estimated With Ordinal Logistic Regression Models

	Coefficient (95% CI) *p-value*			
Language appropriate for use in clinical setting	Poster Only	Patient Vignette	Clinician Vignette	Administrator Vignette
Stigmatizing Terms				
Addict	0.17 (-0.19, 0.52) *0.353*	1.16 (0.78, 1.54) *<0.001*	0.66 (0.30, 1.02) *<0.001*	0.83 (0.48, 1.17) *<0.001*
Substance Abuse	0.36 (-0.00, 0.72) *0.051*	0.96 (0.60, 1.33) *<0.001*	0.83 (0.47, 1.19) *<0.001*	0.99 (0.65, 1.34) *<0.001*
Dirty in Reference to Result from Drug Test	0.06 (-0.32, 0.44) *0.755*	0.53 (0.14, 0.92) *0.008*	0.33 (-0.05, 0.71) *0.087*	0.51 (0.13, 0.88) *0.008*
Clean in Reference to Result from Drug Test	0.49 (0.09, 0.88) *0.016*	0.77 (0.40, 1.14) *<0.001*	0.55 (0.17, 0.93) *0.004*	0.88 (0.52, 1.24) *0.001*
Addicted Baby	0.24 (-0.12, 0.59) *0.189*	0.74 (0.38, 1.11) *<0.001*	0.57 (0.22, 0.92) *0.001*	0.77 (0.42, 1.13) *<0.001*
Non-Stigmatizing Alternatives				
Person with Substance Use Disorder	0.15 (-0.23, 0.53) *0.452*	-0.03 (-0.42, 0.35) *0.871*	0.04 (-0.33, 0.41) *0.833*	0.13 (-0.25, 0.51) *0.502*
Substance Use	0.13 (-0.24, 0.51) *0.487*	0.29 (-0.08, 0.66) *0.129*	0.19 (-0.19, 0.57) *0.318*	0.38 (0.02, 0.75) *0.040*
Negative in Reference to Result from Drug Test	-0.11 (-0.49, 0.27) *0.576*	0.16 (-0.22, 0.54) *0.413*	0.15 (-0.22, 0.52) *0.413*	0.27 (-0.09, 0.63) *0.136*
Positive in Reference to Result from Drug Test	-0.16 (-0.53, 0.21) *0.390*	0.15 (-0.22, 0.51) *0.430*	0.28 (-0.09, 0.65) *0.136*	0.28 (-0.09, 0.65) *0.140*
Baby Born with Neonatal Opioid Withdrawal Syndrome	0.32 (-0.06, 0.69) *0.103*	0.54 (0.16, 0.93) *0.005*	0.53 (0.16, 0.91) *0.005*	0.52 (0.14, 0.89) *0.008*

Ordered logistic regression coefficients display average difference on 5-point Likert scale outcome associated with exposure to message frame relative to no-exposure control group. Model estimates adjust for age (continuous), female gender, prescriber status (physician, nurse practitioner, or physician assistant), and white and not Hispanic or other race or Hispanic ethnicity.

© 2022 Kennedy-Hendricks A et al. *JAMA Network Open*.

eTable 11. Effects of Exposure to the *Medication Treatment Works* Visual Campaign and Narrative Vignettes on Stigma Toward People With Opioid Use Disorder Receiving Medication Treatment Relative to the Nonexposed Control Group Estimated With Ordinal Logistic Regression Models

	Coefficient (95% CI) *p-value*			
	Visual Campaign Only	Visual Campaign + Patient Vignette	Visual Campaign + Clinician Vignette	Visual Campaign + Administrator Vignette
There is a treatment for OUD that is effective for a long period of time.	-0.07 (-0.43, 0.30) *0.728*	0.10 (-0.29, 0.50) *0.605*	-0.22 (-0.60, 0.15) *0.243*	0.10 (-0.29, 0.49) *0.599*
Unwilling to have a person taking medication to treat OUD marry into family	-0.06 (-0.41, 0.28) *0.716*	-0.33 (-0.70, 0.05) *0.089*	-0.20 (-0.54, 0.14) *0.252*	-0.08 (-0.43, 0.27) *0.642*
Unwilling to have a person taking medication to treat OUD as neighbor	0.07 (-0.28, 0.42) *0.688*	-0.39 (-0.75, -0.03) *0.032*	-0.03 (-0.39, 0.32) *0.861*	-0.04 (-0.41, 0.32) *0.813*
Person taking medication with OUD is strong	-0.03 (-0.39, 0.34) *0.881*	0.09 (-0.28, 0.47) *0.626*	0.09 (-0.28, 0.45) *0.643*	-0.03 (-0.40, 0.33) *0.863*
Agree MOUD is more effective than treatment without medication	0.27 (-0.07, 0.61) *0.120*	0.07 (-0.30, 0.44) *0.718*	0.36 (0.01, 0.72) *0.046*	0.19 (-0.17, 0.53) *0.297*
Most people with OUD will, with medication treatment, get well and return to productive lives	-0.26 (-0.61, 0.08) *0.140*	-0.19 (-0.56, 0.18) *0.312*	0.06 (-0.30, 0.43) *0.728*	-0.01 (-0.36, 0.34) *0.954*

Ordered logistic regression coefficients display average difference on 5-point Likert scale outcome associated with exposure to message frame relative to no-exposure control group. Model estimates adjust for age (continuous), female gender, prescriber status (physician, nurse practitioner, or physician assistant), and white and not Hispanic or other race or Hispanic ethnicity.

© 2022 Kennedy-Hendricks A et al. *JAMA Network Open*.

eFigure. *Words Matter* and *Medication Treatment Works* Visual Campaigns

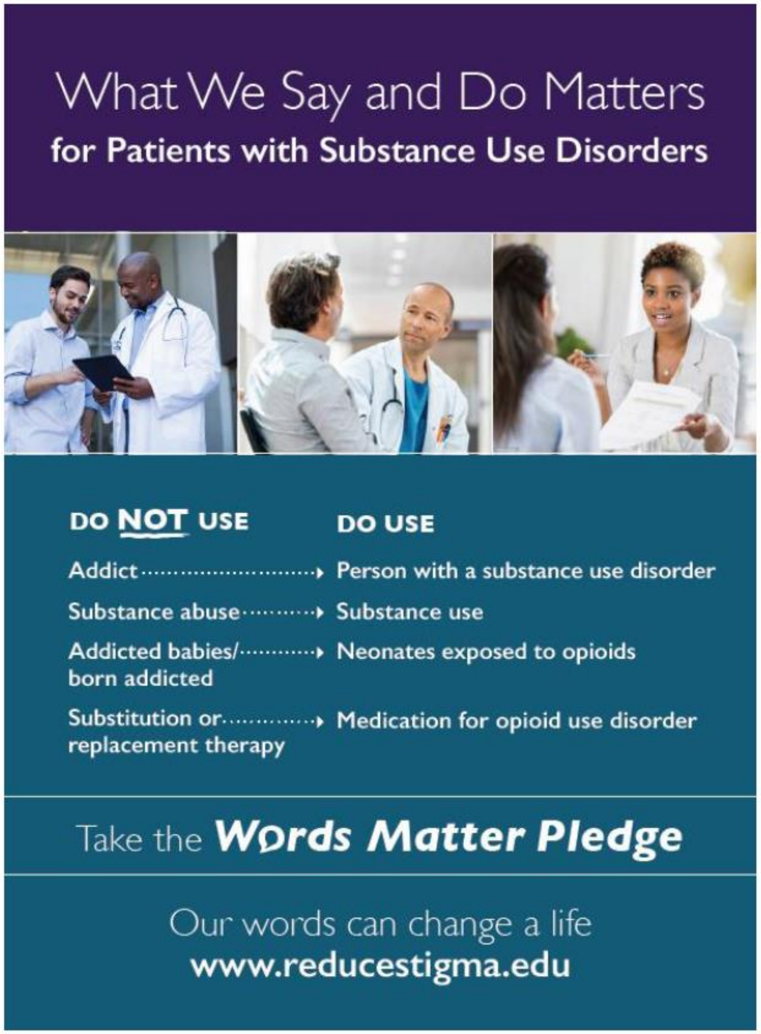

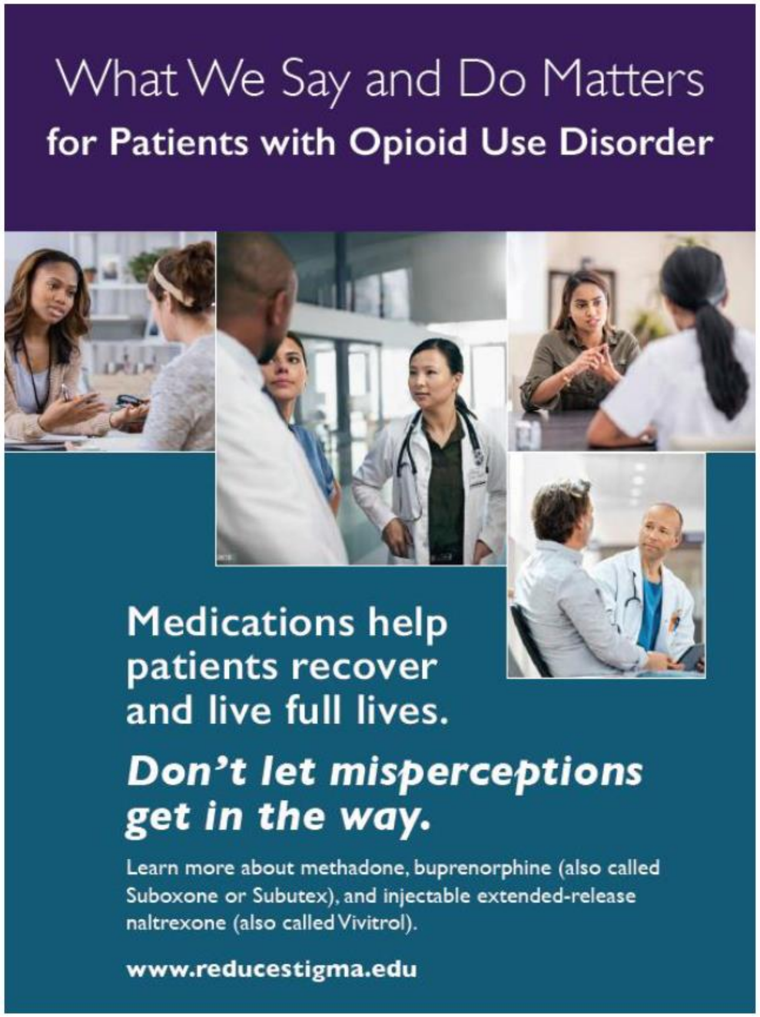

© 2022 Kennedy-Hendricks A et al. *JAMA Network Open*.
