# Supplementary material for: Effect of Exposure to Visual Campaigns and Narrative Vignettes on Addiction Stigma Among Health Care Professionals: A Randomized Clinical Trial
Source: JAMA Netw Open. 2022 Feb 4;5(2):e2146971. doi: 10.1001/jamanetworkopen.2021.46971 (PMC8817201; doi:10.1001/jamanetworkopen.2021.46971)
Supplement: Supplement 3. — Data Sharing Statement [file jamanetwopen-e2146971-s003.pdf]

Data Sharing Statement

Kennedy-Hendricks. Effect of Exposure to Visual Campaigns and Narrative Vignettes on Addiction Stigma Among Health Care Professionals. *JAMA Netw Open*. Published February 04, 2022. doi:10.1001/jamanetworkopen.2021.46971

Data
Data available: No
